# Hyperthermophilic endoglucanase for *in planta* lignocellulose conversion

**DOI:** 10.1186/1754-6834-5-63

**Published:** 2012-08-28

**Authors:** Holger Klose, Juliane Röder, Michele Girfoglio, Rainer Fischer, Ulrich Commandeur

**Affiliations:** 1 Institute for Molecular Biotechnology (Biology VII), RWTH Aachen University, Worringerweg 1, 52074, Aachen, Germany; 2 Institute of Protein Biochemistry, CNR, Via P. Castellino 111, 80131, Naples, Italy; 3 Fraunhofer Institute for Molecular Biology and Applied Ecology (IME), Forckenbeckstrasse 6, 52074, Aachen, Germany

**Keywords:** Sulfolobus solfataricus, Cellulases, Biomass processing, Ionic liquids, Plants

## Abstract

**Background:**

The enzymatic conversion of lignocellulosic plant biomass into fermentable sugars is a crucial step in the sustainable and environmentally friendly production of biofuels. However, a major drawback of enzymes from mesophilic sources is their suboptimal activity under established pretreatment conditions, e.g. high temperatures, extreme pH values and high salt concentrations. Enzymes from extremophiles are better adapted to these conditions and could be produced by heterologous expression in microbes, or even directly in the plant biomass.

**Results:**

Here we show that a cellulase gene (sso1354) isolated from the hyperthermophilic archaeon *Sulfolobus solfataricus* can be expressed in plants, and that the recombinant enzyme is biologically active and exhibits the same properties as the wild type form. Since the enzyme is inactive under normal plant growth conditions, this potentially allows its expression in plants without negative effects on growth and development, and subsequent heat-inducible activation. Furthermore we demonstrate that the recombinant enzyme acts in high concentrations of ionic liquids and can therefore degrade α-cellulose or even complex cell wall preparations under those pretreatment conditions.

**Conclusion:**

The hyperthermophilic endoglucanase SSO1354 with its unique features is an excellent tool for advanced biomass conversion. Here we demonstrate its expression *in planta* and the possibility for post harvest activation. Moreover the enzyme is suitable for combined pretreatment and hydrolysis applications.

## Background

The conversion of lignocellulosic biomass into fuels and commodity chemicals could provide a sustainable alternative to processes based on non-renewable fossil fuel resources. Common strategies to convert lignocellulose into fermentable sugars involve various pretreatment steps followed by enzymatic hydrolysis. Pretreatment methods are usually harsh, involving strong acidic or alkaline solutions, high temperatures and pressures, and the presence of organic solvents [1]. These are features of contemporary chemical processing methods such as *diluted acid hydrolysis*, *ammonia fiber explosion* (AFEX) and *organosolv*, also the use of ionic liquids to dissolve lignocellulose has recently emerged as a promising alternative for biomass pretreatment [2]. Hydrolytic enzymes are commonly produced by microbial fermentation, and despite various improvements in this field the costs are still high [3]. The high energy input required for pretreatment and the expense of enzyme production in microbes means that the conversion of lignocellulosic biomass into fermentable sugars remains economically and environmentally unsustainable [4,5].

Plants can be used as an alternative platform for manufacturing lignocellulolytic enzymes because they can be grown inexpensively on a large scale, which reduces water use and greenhouse gas emissions compared to microbial fermentation [6]. The greatest potential benefit offered by plants is that biomass-degrading enzymes can be produced within the plant biomass itself. Importantly, this approach can only be successful if the enzymatic activity does not inhibit plant growth and development, and biomass pretreatments do not destroy the enzymes [6,7].

Cellulases have been produced in a number of plant species [8,9] and the expressed and isolated recombinant enzymes have been shown to digest cell wall preparations [10,11]. However, the constitutive expression of mesophilic cellulases in plants eventually resulted in abnormal phenotypes such as stunting and deformation [12] reflecting changes in the structure and lower recalcitrance of cell walls [13]. To avoid these detrimental effects, cellulases have been targeted to intracellular compartments so that direct contact with cell wall polysaccharides is avoided until after harvesting and pretreatment, which results in cell disruption [14].

The degradation of plant cell walls during normal growth can also be avoided by the expression of thermophilic cellulases because their temperature optima often lie outside the physiological range for mesophilic plant growth [11,15]. Thermophilic cellulases should also be more compatible with standard pretreatments. For example, *Acidothermus cellulolyticus* endoglucanase E1 was expressed in transgenic tobacco plants that were subjected to AFEX pretreatment, and approximately 30% of the enzyme activity was retained [16]. Several ionic liquids have been evaluated as lignocellulose solvents, and these can be combined with enzymatic hydrolysis although the enzymes must retain their activity in high concentrations of ionic liquids with a water content of only 10–20% [17].

Extremophilic microorganisms can tolerate high temperatures, extreme pH values and strong salt solutions, therefore offering an ideal source of enzymes that convert lignocellulose into fermentable sugars [18,19]. The genome of the hyperthermophilic archaeon *Sulfolobus solfataricus* encodes three different endoglucanases (SSO1354, SSO1949 and SSO2534) [20] that have already shown their potential to perform efficiently under harsh physical and chemical conditions [21-24]. Here we achieved the expression of SSO1354 in tobacco (*Nicotiana tabacum*) and determined its ability to convert plant biomass into fermentable sugars under different pretreatment conditions, offering a promising new approach for the bioconversion of lignocellulose *in planta*.

## Results

### Expression and purification of SSO1354

The *sso1354* gene was amplified from *Sulfolobus solfataricus* genomic DNA without the coding region of the putative leader peptide [23]. Investigation of putative N-glycosylation sites (matching the consensus Asn-X-Ser/Thr) using the web tool “NetNGlyc 1.0 Server” (available at http://www.cbs.dtu.dk/services/NetNGlyc) revealed the presence of 10 potential N-glycosylation sites.

**Figure 1:**
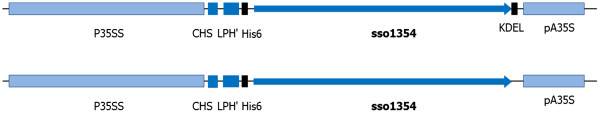
**Schematic representation of SSO1354 expression cassettes from the constructs including an N-terminal His**_**6**_**tag.** The double CaMV promoter (P35SS) and terminator (pA35S) are shown in light blue and the chalcone synthase 5'-UTR (CHS) and codon-optimized leader from the murine antibody peptide mAb24 (LPH) are shown in dark blue. The His_6_ and KDEL coding sequences are indicated in black.

The amplified sequences were flanked by either N-terminal or C-terminal His_6_ tags and an optional C-terminal KDEL sequence for retention in the endoplasmic reticulum (ER) was also included, depending on the primers. These products were transferred to the pTRAkc expression vector. The expression cassette contained the *Cauliflower mosaic virus* (CaMV) double 35SS promoter, the 5'-UTR translational enhancer from the *Petroselinum hortense* chalcone synthase gene and a plant codon optimized LPH signal peptide for secretion to the apoplast (Figure 1).

The expression vectors were introduced into *A. tumefaciens* strain GV3101, which was infiltrated into tobacco leaves. The infiltrated leaves were harvested after 4 days, total soluble protein (TSP) were extracted and analyzed by SDS-PAGE, activity staining and Western blot.

**Figure 2:**
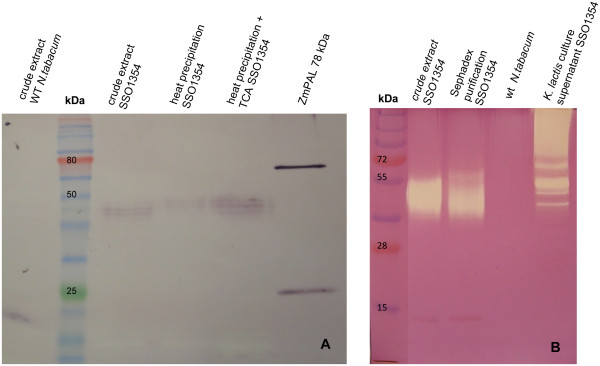
**Western blot (A) after SDS-PAGE and zymography (B) after SDS-PAGE in the presence of 0.15% (w/v) CMC, representing different purification steps following SSO1354 expression in tobacco.** Lanes contain 1 μg of purified SSO1354. The recombinant enzyme was detected with an anti-penta-His primary antibody and an AP-conjugated goat anti-mouse secondary antibody. The multiple bands may represent miscellaneous forms of N-glycosylation.

Initial transient expression tests showed that enzyme variants with a C-terminal His_6_ tag could not be detected in the infiltrated plants. However constructs carrying an N-terminal His_6_ tag either with or without the KDEL sequence were detectable by Western Blot analysis (Figure 2A). The ER-localized variant showed the highest azocarboxymethylcellulase (Azo-CMC) activity per gram leaf material (data not shown) and was selected for expression in transgenic plants and further analysis.

**Figure 3:**
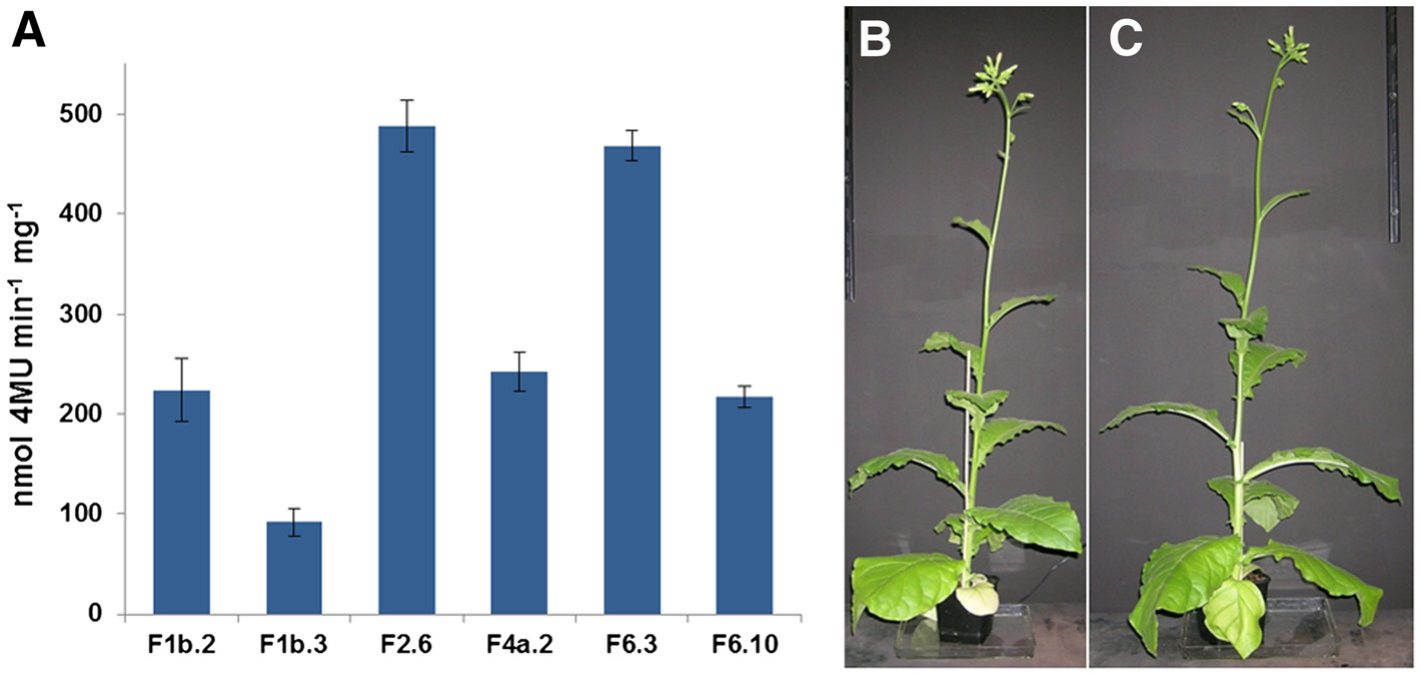
**Expression of SSO1354 in transgenic tobacco lines.** Quantification of expression by 4MUC conversion (**A**). Phenotype of a transgenic tobacco plant expressing SSO1354 (**B**) and wild type SR1 tobacco (**C**), both grown in soil under greenhouse conditions.

T_0_ transgenic plants expressing the ER-tagged enzyme were transferred to soil and maintained in the greenhouse. The leaves were screened for enzyme activity using the substrate SDS-PAGE method, and T_1_ seeds from positive candidates were used to produce the next generation of plants (data not shown). The leaves from the T_1_ plants were tested using the substrate 4-methylumbelliferyl-β-D-cellobioside (4MUC) to determine the activity of the recombinant SSO1354 protein, which ranged from 92 to 488 nmol 4MU min^-1^ mg^-1^ in different transgenic lines (Figure 3A). The morphology and growth behavior of the transgenic plants were no different to those of wild-type SR1 plants (Figures 3B and C).

The recombinant SSO1354 was partially purified from crude plant extracts by thermal precipitation. Heating to 85°C for 10 min caused 95% of the host cell proteins to precipitate whereas SSO1354 remained soluble and active. Reducing sugars in the extract, which could potentially interfere with subsequent enzyme activity assays, were removed by passing the remaining extract through a PD10 desalting column (Figure 2B).

### Biochemical properties and catalytic activity of plant-derived SSO1354

The substrate profile of SSO1354 was determined by testing against a panel of β-glucans. A p-hydroxybenzoic acid hydrazide (PAHBAH) assay was used to measure the release of reducing sugars, showing that SSO1354 was active against CMC (arbitrary value set at 100% activity) and also barley β-glucan (136% relative activity) and lichenan (130% relative activity), but not against PCM3/pachyman. This suggests that SSO1354 has a β-1,4 hydrolytic mode of action. The enzyme showed no significant activity on Avicel®, a microcrystalline cellulose, or on oat xylan.

**Figure 4:**
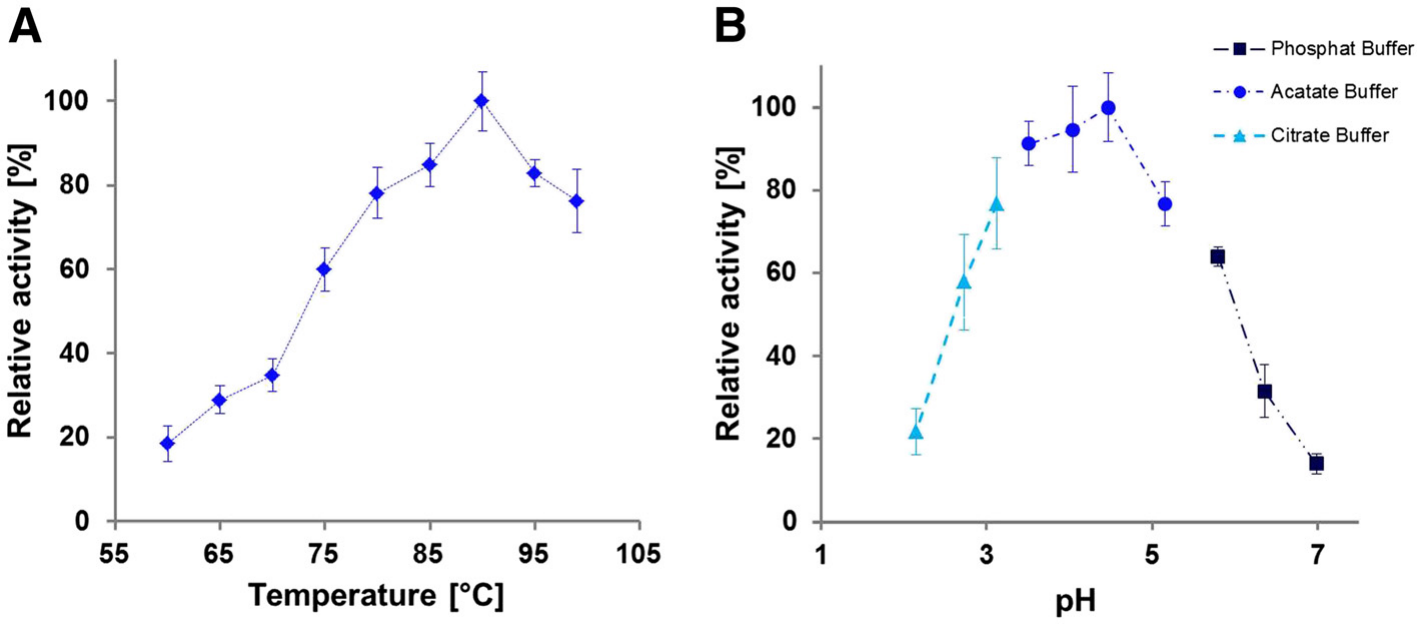
**Influence of temperature(A)and pH (B) on SSO1354 activity.** Conversion of Azo-CMC was measured at 60–99°C. The maximum activity measured at 90°C was set at 100%. Influence of pH on SSO1354 activity was measured by Azo-CMC conversion at pH values between 2.0 and 7.0. The maximum activity measured at pH 4.5 was set at 100%.

**Figure 5:**
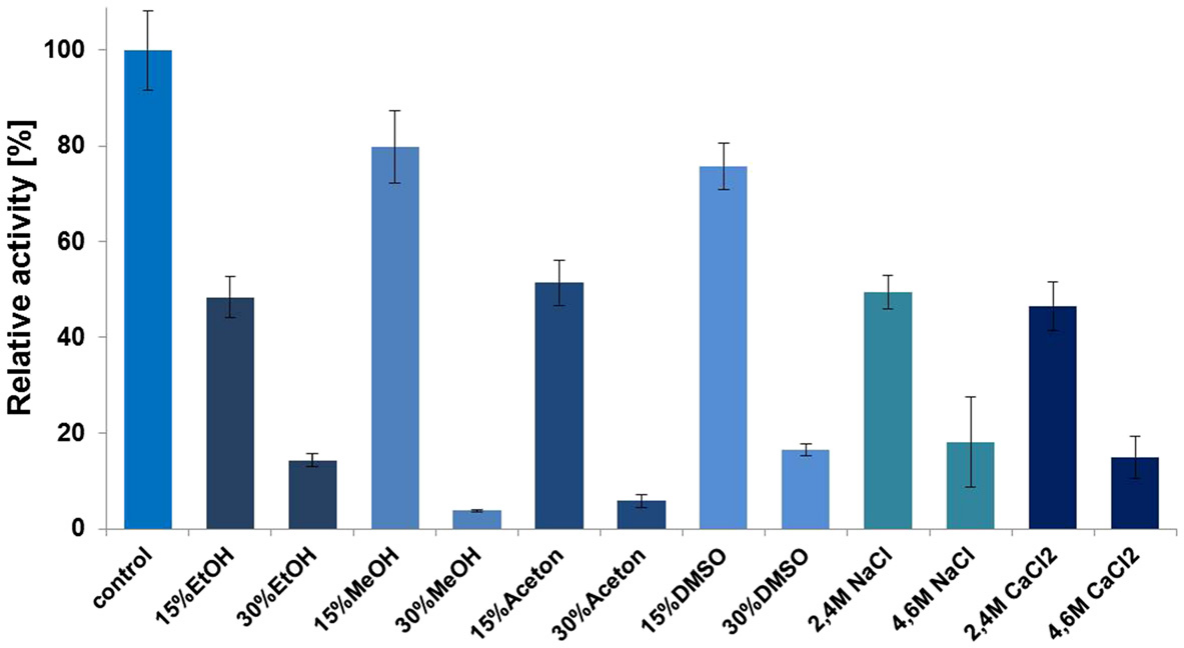
**Influence of solvents and different salt concentrations on SSO1354 activity.** Activity was measured using the Azo-CMC assay at optimal conditions (90°C, pH 4.5) by adding different concentrations of salts and solvents. SSO1354 activity in the absence of salts and solvents was set at 100%.

Further analysis using an Azo-CMC assay showed that SSO1354 has an optimum temperature at 90°C, although it retained 79% residual activity at 99°C. The activity was intensely reduced at lower temperatures, falling to 24% residual activity at 70°C. The optimal pH was 4.5, although the enzyme retained 58% residual activity at pH 2.7 and 62% residual activity at pH 6.5 (Figure 4). There was only minimal activity below pH 2.2 and above pH 7.0. At optimal conditions (90°C, pH 4.5) the enzyme retained 50% of its activity after incubation for 100 min. SSO1354 also tolerated high salt concentrations, with 49.5% residual activity in the presence of 2.4 M NaCl, and 46.5% residual activity in the presence of 2.4 M CaCl_2_ (Figure 5).

SSO1354 activity was inhibited by 15% (v/v) solutions of both protic solvents (capable of donating protons) such as methanol and ethanol, and aprotic solvents such as acetone and dimethylsulfoxide (DMSO). The enzyme retained 79% residual activity in the presence of methanol, 75% in the presence of DMSO, 51% in the presence of acetone and 48% in the presence of ethanol. Increasing the solvent concentration to 30% (v/v) exacerbated the effect, with methanol and acetone causing near-complete inhibition, DMSO leaving a residual activity of 14% and ethanol leaving a residual activity of 16%.

### SSO1354 activity in ionic liquids

As discussed above, SSO1354 cannot hydrolyze crystalline cellulose or cell wall polysaccharides efficiently in aqueous solutions, but concentrated solutions (80–90% v/v) of ionic liquids such as 1-ethyl-3-methylimidazolium acetate (EMIM-Ac) and 1,3-dimethylimidazolium dimethylphosphate (MMIM-DMP) are able to dissolve crystalline cellulose like Avicel® and cell wall polysaccharides, making them more accessible to hydrolytic enzymes.

**Figure 6:**
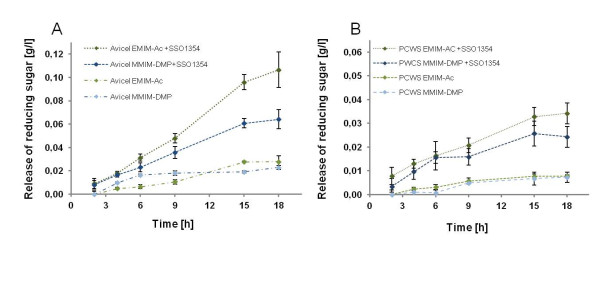
**Activity of SSO1354 on (A) 0.5% (w/v) Avicel® and (B) tobacco-derived cell wall polysaccharides (PCWS) dissolved in 80% (v/v) EMIM-Ac or MMIM-DMP.** Assays were carried out at 90°C. The amount of reducing sugar was measured using the PAHBAH assay.

We therefore tested the activity of SSO1354 on Avicel® that had been dissolved in 80% (v/v) EMIM-Ac and MMIM-DMP by measuring the release of reducing sugars. Sugars released by spontaneous hydrolysis in the absence of the enzyme were measured in control reactions and subtracted from the test reactions, resulting in corrected release rates of 5.5 mg l^-1^ h^-1^ in EMIM-Ac and 3.5 mg l^-1^ h^-1^ in MMIM-DMP (Figure 6A). We also found that cell wall polysaccharides dissolved in EMIM-Ac and MMIM-DMP were hydrolyzed by SSO1354 with a release rate of 1.8 mg l^-1^ h^-1^ for EMIM-Ac and 1.2 mg l^-1^ h^-1^ for MMIM-DMP (Figure 6B).

## Discussion

The conversion of lignocellulosic biomass into fermentable sugars is an important step in the production of second-generation biofuels. A typical approach involves the enzymatic hydrolysis of cellulose using microbial β-glucanases, but the efficiency of saccharification is limited by the accessibility of the substrate, which must therefore be pretreated [1]. Common cellulase preparations are derived from mesophilic fungi and have an optimal activity at 50°C and pH 4–5 [25]. In contrast, standard biomass pretreatment methods are performed under harsher conditions that would invariably denature these enzymes, which means they can only be added after pretreatment [1]. The production of cellulases directly in the biomass targeted for conversion would be more economically and environmentally sustainable than microbial fermentation, but commonly used mesophilic enzymes would be incompatible with current pretreatment regimens [4,26]. Constitutively expressed and secreted mesophilic cellulases can also inhibit normal plant growth, by reducing the integrity of the cell wall and therefore leaving the plant vulnerable to environmental stresses [13]. One solution to this problem is the sequestration of recombinant cellulases into subcellular compartments with no direct access to cell wall components, and previous studies have demonstrated the feasibility of this approach [9,27]. The expression of cellulases from hyperthermophilic organisms is a promising alternative because the temperature optima of these enzymes would ensure normal plant growth and development at physiological temperatures and enzyme survival and activity under conventional pretreatment conditions.

The *sso1354* gene from *S. solfataricus* encodes an endo-β-glucanase from GH family 12 [20] and does not contain a cellulose binding module (CBM). This enzyme is active at high temperatures and at low pH conditions, and it is one of the first enzymes known to retain their activity in high concentrations of ionic liquids [23]. These features make SSO1354 an ideal candidate for enzymatic biomass conversion directly in plants.

We demonstrated that SSO1354 can be functionally expressed in tobacco plants by targeting the protein either for secretion into the apoplast or for retention in the ER. The addition of a His_6_ tag to the N-terminus and an optional C-terminal KDEL sequence had no adverse effects on enzyme activity. The inclusion of the KDEL sequence resulted in different detectable forms of the protein, with molecular weights between 40 and 50 kDa. The predicted molecular weight of the protein was 38 kDa, and the discrepancy may reflect the presence of glycan residues on one or more of the 10 putative N-glycan acceptor sites.

Analysis of the phenotype revealed no significant difference between wild-type tobacco and transgenic lines producing SSO1354 underlining that the enzyme stays inactive at plant growth conditions. The activity of plant-derived SSO1354 reached a maximum of 0.5 μmol 4MU min^-1^ mg^-1^, which represents a yield of 1.1% TSP. This yield is lower than has been reported for other cellulolytic enzymes in plant expression systems [9,28]. However, it may be possible to improve the yields by various means e.g. codon optimization.

We confirmed the known features of SSO1354 [23,24] but we also discovered some novel characteristics, such as the efficient hydrolysis of substrates with β-1,4-linked glucose units. There was no hydrolysis of crystalline cellulose or substrates lacking β-1,4 linkages, nor did we observe the hydrolysis of substrates like oat xylan, which comprises β-1,4-linked xylose, even though such activity has been reported previously [24]. This confirms that the enzyme is a strict endo-β-1,4-glucanase. The enzyme has a temperature optimum of 90°C, which is the highest reported temperature for any cellulase expressed in plants and one of the highest in any heterologous expression system [22,29,30]. The activity of the enzyme drops sharply as the temperature declines, displaying only 25% activity at 70°C and virtually no activity at temperatures below 30°C (data not shown), suggesting there would be no hydrolysis of plant cellulose under normal plant growth conditions. This is supported by the normal morphological phenotype of the transgenic plants (Figures 3B and C) and suggests that post-harvest cellulose hydrolysis could be induced by a temperature shift.

SSO1354 is also advantageous because it tolerates a relatively broad pH range (2.7–6.5, retaining at least 60% residual activity) whereas SSO1949 from the same species has a sharp optimum at pH 1.8 [22]. Under the optimal conditions (pH 4.5 and 90°C), SSO1354 retains 85% activity after 1 h of incubation. In addition to its remarkable thermostability and pH tolerance, SSO1354 also tolerates moderate levels of organic solvents and high levels of salt.

Ionic liquids are very efficient in dissolving crystalline cellulose, however they are still expensive [17,31]. Promising results of their recycling have been published recently [31]. A unique feature of SSO1354 is its activity in ionic liquids such as EMIM-Ac and MMIM-DMP. SSO1354 was able to produce 5.5 mg l^-1^ h^-1^ of reducing sugars from Avicel® dissolved in EMIM-Ac, which is low compared to other soluble substrates but still highly promising for future optimization. The hydrolysis rate was 3.5 mg l^-1^ h^-1^ in MMIM-DMP, showing this is a less suitable ionic liquid for SSO1354-catalyzed biomass hydrolysis.

Although testing SSO1354 on artificial substrates confirmed its activity under a range of conditions, plant biomass is a complex substrate with different components such as cellulose, hemicelluloses and lignin, the latter acting as a significant inhibitor of cellulases (hence the need for pretreatment) [32]. To investigate the activity of SSO1354 against such complex substrates, we exposed the enzyme to cell wall polysaccharides from tobacco leaves. This substrate is more heterogeneous than crystalline cellulose but it has a ~0.8% (dry weight) lignin content and is therefore less complex than material derived from tobacco roots or stems [33]. SSO1354 showed no significant activity against cell wall polysaccharides in aqueous suspension, as determined by the release of reducing sugars, but when the substrate was dissolved in ionic liquids the results were more impressive. The enzyme released 1.8 mg l^-1^ h^-1^ of reducing sugars in EMIM-Ac and 1.2 mg l^-1^ h^-1^ in MMIM-DMP. These data indicate the possibility of combined pretreatment and enzymatic hydrolysis.

## Conclusion

The use of plants as an enzyme production system and subsequent utilization requires two important criteria to be satisfied. The enzyme must not interfere with cell wall synthesis or integrity and it must tolerate the extreme conditions found in standard pretreatment processes. Hyperthermophilic cellulases like the endoglucanase SSO1354 from *S. solfataricus* combine both features. This study demonstrates the successful production of inactive SSO1354 at normal growth conditions followed by the induction of enzyme activity at higher temperatures. To our knowledge, SSO1354 is the first enzyme expressed *in planta* that remains active in ionic liquids such as EMIM-Ac and MMIM-DMP, which are used for biomass pretreatment. Plants have already been developed as a powerful platform for the expression of biomass-processing enzymes, but the use of enzymes that function optimally under harsh pretreatment conditions opens the way to develop combined pretreatment and enzymatic hydrolysis strategies for the efficient conversion of lignocellulosic plant biomass into fermentable sugars.

## Methods

### Plant material and bacterial strains

Tobacco plants (*Nicotiana tabacum* L. cv. Petit Havana SR1) were cultivated in the greenhouse with a 16-h photoperiod (5000–10,000 Lux), 70% relative humidity and 25/22°C day/night temperature. For transient expression and plant transformation *Agrobacterium tumefaciens* strain GV3101::pMP90RK (Gm^R^ Km^R^ Rif^R^) was used [34].

### Cloning

We used binary vectors pTRAkc-AH and pTRAkc-ERH, both of which carry the CaMV 35SS double promoter for constitutive transgene expression [35]. The coding sequence of the SSO1354 catalytic domain (AAK41590.1, EMBL-CDS) was amplified by PCR from *S. solfataricus* P2 genomic DNA (kindly provided by Raffaele Cannio, Institute for Microelectronics and Microsystems, Naples, Italy) using the primers sso1354-fw-*Bsp*HI (5’-AAT CAT GAA GCA GTC TCT CAG CGT TAA ACC CG-3’) and sso1354-rv-*Not*I (5’-AAG CGG CCG CGA GGA GAG TTT CAG AAA AG-3’) to generate the product sso1354-His_6_ with a C-terminal His_6_ tag. The purified fragment was transferred to the pCR2.1 vector (Invitrogen) by TA-cloning to generate construct pCR2.1-SSO1354. This was digested with BspHI and NotI and the released cassette was inserted into the pTRAkc-AH and pTRAkc-ERH vectors (previously digested with *Nco*I and *Not*I). An analogous process, substituting primers sso1354-fw-*Bsp*HI-His6 (5’-TCA TGA AAC ATC ACC ATC ACC ATC ACG CGG CCG CTC AGT CTC TCA GCG TTA AAC CCG T-3’) and sso1354-rv-*Xho*I (5’-AAC TCG AGT TAG AGG AGA GTT TCA GAA AAG-3’) or sso1354-rv-KDEL-*Xho*I (5’-AAC TCG AGC TAG AGC TCA TCT TTG AGG AGA GTT TCA GAA AAG-3’), was used to generate constructs with an N-terminal His_6_ tag, with or without a C-terminal KDEL signal.

### Agroinfiltration and generation of transgenic plants

Electrocompetent *Agrobacterium tumefaciens* cells [36] were transformed with either pTRAkc-AH or pTRAkc-ERH and were selected on YEP medium plates (10 g l^-1^ Bacto Tryptone, 10 g l^-1^ yeast extract, 5 g l^-1^ NaCl, 15 g l^-1^ agar, pH 7.0) supplemented with kanamycin (50 mg ml^-1^), rifampicin (50 mg ml^-1^) and carbenicillin (100 mg ml^-1^).

Colonies were transferred to liquid YEP medium containing kanamycin (50 mg ml^-1^), rifampicin (50 mg ml^-1^) and carbenicillin (100 mg ml^-1^) for 36–40 h (26°C, 180 rpm) for selection. The suspensions were supplemented with 10 μM acetosyringone, 10 mM MES (pH 5.6) and 10 mM glucose, and the bacteria were incubated for another 20 h. The OD_600_ of the culture was adjusted to 1.0 with 2x infiltration medium (0.86% MS salts, 10% sucrose, 0.36% glucose, pH 5.6) and the adjusted suspension was supplemented with 200 μM acetosyringone prior to incubation for 45 min at room temperature.

Transgenic tobacco lines were generated using the leaf disc transformation method [37]. T_0_ plants were grown on MS medium containing 100 mg l^-1^ kanamycin and 200 mg l^-1^ Claforan® (cefotaxime) before transfer to soil in the greenhouse. The T_0_ plants were self-pollinated to generate T1 seeds.

### Protein extraction and purification

Infiltrated leaves were ground in liquid nitrogen and homogenized in phosphate buffered saline (PBS; pH 7.0) supplemented with 1 mM phenylmethylsulfonylfluoride (PMSF). The extract was centrifuged at 15,000 × *g* for 20 min at 4°C followed by filtration to remove suspended particles from the extract. Bulk proteins were removed by heat precipitation at 85°C for 10 min and the recombinant enzyme was separated from small molecules such as sugars by gel filtration (PD-10 Columns, GE Healthcare). Total protein levels were determined using the Bradford method [38] with bovine serum albumin (Roth) as the standard.

### Substrate SDS-PAGE and Western blot

Protein samples were separated by SDS-PAGE in a 12% polyacrylamide gel containing 0.15% (w/v) carboxymethylcellulose (CMC). The proteins were then renatured by washing the gels twice at room temperature for 15 min with 50 mM sodium acetate buffer (pH 4.8) containing 20% (v/v) propan-2-ol followed by washing twice for 30 min with the same buffer containing no propan-2-ol.

The samples were incubated in sodium acetate buffer at 85°C for 20 min and then in 50 mM Tris–HCl (pH 7.5) for 30 min at room temperature to stop the reaction. The gels were stained for 30 min in 0.1% (w/v) Congo Red (Sigma) and destained in 1 M NaCl. To achieve a higher contrast between degradation and non-degradation areas, the gel was incubated after destaining with 0.5% (v/v) acetic acid.

For Western blot analysis, proteins separated by SDS-PAGE were electro-transferred (60 min, 250 mA) to nitrocellulose membranes, blocked for 1 h at room temperature with 5% (w/v) skimmed milk dissolved in PBS, and probed with a monoclonal antibody against penta-His (Qiagen). After washing, the signal was detected with a secondary monoclonal alkaline phosphatase-conjugated goat anti-mouse antibody (Dianova) and visualized with nitroblue tetrazolium and 5-bromo-4-chloro-3-indolyphosphate (NBT/BCIP; Roth).

### Enzymatic assays

Endoglucanase assays using the substrate 4MUC (Sigma Aldrich) were carried out as described in 50 mM sodium acetate buffer (pH 4.5) containing 0.5 mM 4MUC [39] The conversion rate was calculated against 4MU standards ranging from 0.05 to 2 nmol. For the calculation of the expression rate in % of TSP, measured activity values were compared to a purified recombinant SSO1354 preparation.

The soluble chromogenic substrate AZO-CM-Cellulose (Megazyme) was used to determine the temperature, pH and salt tolerance of the recombinant enzyme in aqueous solutions as previously described [40]. Thermotolerance (70–100°C) was determined using 50 mM acetate buffer (pH 4.5), pH tolerance was determined at 90°C using 50 mM citrate buffer (pH 2.0–3.5), 50 mM acetate buffer (pH 3.5–5.5) and 50 mM phosphate buffer (pH 5.5–7.0). Salt tolerance was determined using 50 mM acetate buffer (pH 4.5) at 90°C supplemented with up to 4.6 M NaCl. We took five replicates at each measurement point.

### Determination of activity in ionic liquids

We tested enzyme activity in 1-ethyl-3-methylimidazolium acetate (EMIM-Ac) and 1,3-dimethylimidazolium dimethylphosphate (MMIM-DMP), both diluted to 80% (v/v) in water, using either Avicel® or tobacco-derived cell wall polysaccharides as the substrate (0.5% w/v). The aqueous-buffered enzyme was added to a final concentration of 0.2 g l^-1^ and 20% (v/v) buffer in the reaction mixture. The reaction mixture was incubated in an Eppendorf Thermomixer at 1000 rpm and 90°C and samples were taken at intervals to determine reducing sugar levels.

The reaction was stopped by cooling each sample and the cellulose was precipitated by adding one volume of water followed by centrifugation (15,000 × *g* for 2 min). Three phenol-chloroform (1:1) extractions steps and one chloroform extraction were carried out to separate the hydrolysis products and proteins from the ionic liquid. The reducing sugar content was measured using the p-hydroxybenzoic acid hydrazide (PAHBAH) assay by mixing one volume of sample with two volumes of PAHBAH reagent [41]. The solution was incubated at 100°C for 10 min and absorbance was measured at 410 nm. Defined concentrations of glucose were used for calibration. Five replicates were measured at each time point.

### Preparation of tobacco cell wall polysaccharides

Tobacco cell wall polysaccharides were prepared as previously described [10]. Briefly, tobacco leaves were harvested and ground to powder under liquid nitrogen followed by two washes with 25 ml distilled water and centrifugation at 20,000 × g, 4°C. The insoluble cell wall material was washed several times with methanol-chloroform (1:1) until the green color disappeared completely. The organic solvent was then removed by evaporation and the dried powder was used for enzymatic assays in ionic liquids.

## Abbreviations

4MU: 4-Methylumbelliferone; 4MUC: 4-Methylumbelliferyl-β-D-cellobioside; AFEX: Ammonia fiber explosion; BCIP: 5-Bromo-4-chloro-3'-indolyl phosphate p-toluidine; CBM: Cellulose binding module; CMC: Carboxymethylcellulose; DMSO: Dimethylsulfoxide; EMIM-Ac: 1-Ethyl-3-methylimidazolium acetate; ER: Endoplasmic reticulum; GH: Glycoside hydrolase; MES: 2-(N-morpholino)ethanesulfonic acid; MMIM-DMP: 1,3-Dimethylimidazolium dimethylphosphate; NBT: Nitroblue tetrazolium chloride; PAHBAH: p-Hydroxybenzoic acid hydrazide; PBS: Phosphate buffered saline; PMSF: Phenylmethylsulfonylfluoride; SDS: Sodium dodecylsulfate; TSP: Total soluble protein; Tris: Tris(hydroxymethyl)aminomethane; UTR: Untranslated region; YEP: Medium containing Yeast extract and peptone.

## Competing interests

The authors declare that they have no competing interests.

## Authors’ contributions

HK designed and carried out the experiments, analyzed results and wrote the manuscript. JR cloned the SSO1354 gene. MG assisted in the experimental design and reviewed the manuscript. RF and UC coordinated the study and reviewed the manuscript. All authors read and approved the final manuscript.
